# Supplementary material for: WordCommentsAnalyzer: A windows software tool for qualitative research
Source: F1000Res. 2018 Sep 4;7:536. Originally published 2018 May 3. [Version 2] doi: 10.12688/f1000research.14819.2 (PMC6137411; doi:10.12688/f1000research.14819.2)
Supplement: Supplementary file 3 [file f1000research-7-17614-s0002.tgz › b2a79496-4ada-47c4-9682-a99ce867836d.zip!/Eight Iranian Health Journals/Archives of Rehabilitation_71.docx]

© Archives of Rehabilitation. This work is licensed under CC BY-NC 4.0 (https://creativecommons.org/licenses/by-nc/4.0/).

Volume & Issue: دوره 1 - شماره 3

Title: بررسی نقش تسهیل کننده روش کرانیوساکرال تراپی همراه با روش بوبت در کاهش اسپاستیسیته اندام تحتانی کودکان دایپلژی اسپاستیک 3 تا 8 ساله

http://rehabilitationj.uswr.ac.ir/article-1-523-fa.html

هدف: هدف از انجام این کار کارآزمایی بالینی، بررسی نقش تسهیل کننده روش کرانیو ساکرال تراپی در کاهش اسپاستی سیته اندام تحتانی کودکان دایپلژی اسپاستیک بوده است. روش بررسی: این بررسی در جامعه آماری 100 کودک فلج مغزی صورت گرفته و 36 کودک دایپلژ ی اسپاستیک در دامنه سنی 3-8 سال در دو گروه 18 نفری کنترل و مورد، تحت مطالعه قرار گرفتند. افراد هر گروه طی 30 جلسه درمانی به صورت 3 جلسه در هفته و هر جلسه به مدت یک ساعت تحت درمان قرار گرفتند. در گروه مورد ابتدا تکنیک های عمومی و ده گانه و سپس تکنیکهای موضعی کرانیوساکرال تراپی انجام می شد، سپس مطابق روش بوبت تحت درمان قرار می گرفتند اما در گروه شاهد تنها از روش بوبت استفاده می شد. ارزیابی تونوس عضلانی به وسیله معیارModifed Ashworth دامنه حرکتی غیر فعال به وسیله گونیامتری، مرحله رشد حرکتی توسط معیار بوبت انجام گرفت. کلیه کودکان در طی سه ماه، دوبار (یک بار پیش از شروع درمان و یکبار در پایان مدت تعیین شده) مورد ارزیابی قرار گرفتند و نتایج حاصله در هر گروه توسط آزمون آماری «تی زوجی» (2) و بین دو گروه توسط آزمون آماری «تی مستقل» با یکدیگر مقایسه شد،در مورد نتایج حاصله نیز از آزمون «همبستگی» (4) به منظور بررسی همبستگی ها استفاده گردید، و برای بررسی ارتباط بین جنس و گروه های مورد تحقیق، آزمون «کای .دو» (5) مورد استفاده قرار گرفت. یافته‌ها: نتایج حاصله نشان دهنده بهبودی معنی دار در هر دو گروه بود؛ اما در گروه مورد، کاهش هایپر تونسیته اداکتورهای رانی و پلانتارفلکسورهای مچ پا از گروه شاهد بود؛ همچنین در گروه مورد افزایش دامنه حرکتی غیر فعال بداکشن رانی و دورسی فلکشن مچ پا بیشتر از گروه شاهد بود و نیز ارتباط معنی داری بین افزایش دامنه حرکتی فعال ابداکش رانی و بهبود مرحله رشد حرکتی با کاهش هایپر تونیسیته اداکتورهای رانی دیده شد. نتیجه‌گیری:  تمام موارد بالا در مجموعه 5% بوده و عواقب بالینی O.T. ذکر شده است.

Title: تاثیر یک دوره بازتوانی قلبی بر توانایی جنسی بیماران مبتلا به سکته قلبی

http://rehabilitationj.uswr.ac.ir/article-1-524-fa.html

هدف: یکی از شکایات معمول بیماران استکمیک قلب ناتوانی جنسی(1) است. بعد از اولین انفارکتوس میوکارد معمولا میزان فعالیت جنسی در بیماران کاهش می یابد. این کاهش در فعالیت جنسی، در بیماران مذکر و مونث تفاوتی ندارد. شایعترین مشکلات جنسی در بیماران کرونری کاهش یا از بین رفتن میل جنسی (2)، ناتوانی جنسی و انزال زودرس یا دیررس است. بازتوانی، در کاهش بسیاری از عوارض همراه بیماریهای قلبی مثل اضطراب و استرس نقش موثری داشته است. هدف از این تحقیق، بررسی اثر بازتوانی قلبی روی میزان فعالیت جنسی بیماران است. روش بررسی: در این مطالعه 60 بیمار شرکت داشتند، افراد شرکت کننده همگی مذکر بودند و سن آنها بین 35 تا 65 سال بود. سی نفر در گروه کنترل و سی نفر در گروه مورد قرار گرفتند. کلیه بیماران مبتلا به انفارکتوس میوکارد بودندو یک ماه از زمان بستری شدن آنها در CCU گذشته بود. گروه مورد از بیمارانی تشکیل شده بود که جهت بازتوانی قلبی به قسمت بازتوانی مرکز تحقیقات قلب و عروق اصفهان معرفی شده بودند. گروه شاهد بیمارانی بودند که بعداز ترخیص از CCU تحت بازتوانی قلب قرار نگرفته بودند. قبل از شروع مطالعه ، پرسشنامه های مربوطه برای هر دو گروه پرشدند، سپس بازتوانی گروه مورد آغاز شد. میزان اضطراب و افسردگی توسط پرسشنامه 90 SCI بررسی شد. سنجش فعالیت جنسی توسط پرسشنامه ای که شامل 10 پرسش بود، انجام شد. پرسشنامه ها، قبل و بعد از بازتوانی توسط یک پرسشگر تکمیل می شدند. یافته‌ها: محاسبات آماری توسط نرم افزار SPSS انجام شد. ابتدا میانگین نمره های هر مورد، قبل و بعد از بازتوانی محاسبه شد. میانگین تغییرات برای هرکدام از موارد تحت بررسی ، توسط تی تست زوجی (3) در گروه مورد و شاهد جداگانه مقایسه شدند. میانگین تغییرات به دست آمده در گروه های مورد و شاهد توسط تی تست مستقل (4) مقایسه شدند. در کلیه موارد تحت بررسی میانگین نمرات قبل و بعد از بازتوانی به طور معنی داری کاهش یافته بو غیر از لیبیدو که افزایش نمره P<0/05 داشت در مقایسه گروه بازتوانی با گروه شاهد، فقط در مورد افسردگی و میل جنسی و ناتوانی جنسی این تغییرات تفاوت معنی داری داشتند، P<0/05 در مورد زودرس (5) و اضطراب تفاوتها معنی دار نبودند. نتیجه‌گیری: این یافته ها نشان می دهند که احتمالا بازتوانی قلبی روی ناتوانی جنسی تاثیر مثبت دارد. در هر دو گروه، بهبود قابل توجهی در وضعیت بیماری مشاهده شده است. علت معنی دار نبودن تفاوت بین گروه مورد شاهد در سایر موارد مربوط به کم بودن تعداد نمونه باشد.

Title: بررسی سندرم Q-T طولانی در کودکان با کری حسی-عصبی شهر تهران

http://rehabilitationj.uswr.ac.ir/article-1-525-fa.html

هدف: سندرم QT طولانی با منشا ناشناخته، بیماری غیر شایعی است که باعث تغیرات غیر طبیعی دپلاریزاسیون در الکترو کاردیوگرام بیماران می شود و به صورت سنکوپ یا اختلال هشیاری ناشی از تاکیکاردی یا فیبریلاسیون بطنی رخ می دهد. این سندرم می‌تواند با کری حسی – عصبی مادرزادی، به صورت اتوزومال مغلوب انتقال یابد (سندرم ژرول لانگ نیلسن). هدف از این مطالعه، بررسی یافتن الکتروکاردیو گرامهای غیر طبیعی در کودکان با کری حسی – عصبی در جمعیت شهر تهران بود؛ کاری که تاکنون در ایران نگرفته است. روش بررسی: از میان 1190 بیمار با ناشنوایی، 799 بیمار کری حسی. عصبی داشته اند، سن متوسط آنان 13+3/8 سال و %63 دختر، %37 پسر هستند. سابقه خانوادگی ناشنوایی در %19 موارد یافت می شد. انحراف محور در 56 مورد (%7) اختلالات هدایتی در 12 مورد (1/5درصد)، سندرم wpw در 2 مورد (0/25 درصد)، برادیکاردی سینوسی در 4 مورد (0/5) درصد  و آریتمی-سینوسی در 3 مورد (0/38درصد) مشاهده شد. فاصله QT در این جمعیت با متوسط 32msec و انحراف معیار 28/9 با حداقل 200ms و حداکثر 500ms و QT اصلاح شده با متوسط 383 و انحراف معیار 29/3 تعیین شد (حداقل 232ms و حداکثر 527ms). یافته‌ها: سندرم QT طولانی در 4 بیمار (0/5درصد) 3 دختر و یک پسر، یافت شد. از این 4 بیمار، 2 مورد کری کامل و 2 مورد کاهش شنوایی عمیق داشتند. در هیچ یک از بیمارین باکری خفیف، افزایش فاصله Q-T مشهود نبود. تنها یکی از بیماران سمپتوماتیک بود که سالها با تشخیص صرع تحت درمان دارویی قرار گرفته بود. نتیجه‌گیری: این یافته ها، وجود سندرم Q-T طولانی در بیماران با کاهش شنوایی حسی، عصبی را نشان می دهد. بنابراین، گرفتن الکتروکاردیوگرام برای تمامی این بیماران توصیه می شود تا از آریتمی های بطنی و مرگ ناگهانی متعاقب آن جلوگیری به عمل آید.

Title: بررسی اثر تحریکات الکتریکی بر روی قدرت و تحمل عضلانی از دیدگاه الکترومیوگرافی

http://rehabilitationj.uswr.ac.ir/article-1-526-fa.html

هدف: تقویت عضلات ارادی و نیز افزایش تحمل عضلانی از اهداف مهم برنامه های فیزیوتراپی هستند و بدین منظور، روش‌های متعدد تمرین درمانی و استفاده از تحریکات الکتریکی مورد مطالعه قرار گرفته و از هرکدام، نتایج سودمندی حاصل شده است. با این حال، پیشبرد بازده روش‌های متعدد و رسیدن به روش‌های مفیدتر در این زمینه، همواره راه تحقیقات این بخش از فیزیوتراپی را باز گذاشته است. در این مقاله، قصد ما ارائه نتایج حاصل از یک رژیم تقویت و افزایش تحمل عضلانی در مردان سالم در گروه سنی 21-31 سال با استفاده از جریانهای تداخلی است. روش بررسی: نمونه 34نفری مورد مطالعه به روش غیر تصادفی از جامعه دانشجویان دانشکده علوم توانبخشی در تهران انتخاب شدند و برنامه مورد مطالعه تحریک الکتریکی و گرفتن 15انقباض حداکثر و ایزومتریک 25ثانیه ای با استراحت 35ثانیه به مدت 15دقیقه به طور روزانه روی عضله دو سر بازویی سمت راست با فرکانس 40-60 هرتز و فرکانس حامل 2000 هرتز بود. برای ارزیابی تاثیر برنامه دو هفته ای پارامترهای فعالیت الکتریکی عضله و آنتروپومتریک مدنظر بوده است. یافته‌ها: آزمون آماری «تی زوجی» تغییر معنی داری در مدت موج بر انگیخته عضلانی ، حداکثر فعالیت الکتریکی عضله در انقباض ایزومتریک ارادی، تحمل عضلانی، قطر استراحت بازو ، قطر حداکثر انقباض عضلانی نشان داده بود، ولی تغییر دامنه موج برانگیخته عضلانی معنی دار نبود. نتیجه‌گیری: نتایج این تحقیق حاکی از آن است که در افراد سالم می توان از رژیم تحریک الکتریکی به منظور تقویت عضلات ارادی و افزایش تحمل عضلانی استفاده کرد.

Title: بررسی اپیدمیولوژیک بیماری اسکلروز منتشر در 187 بیمار بستری شده در مراکز آموزشی درمانی تهران در سال 1377

http://rehabilitationj.uswr.ac.ir/article-1-527-fa.html

هدف: اسکلروز منتشر بیماری مزمن است که بیشتر ، زنان را گرفتار می کند. بروز بیماری از سن نوجوانی شروع و به تدریج افزایش یافته، بعد از 35 سالگی کاهش پیدا می کند. توزیع چغرافیایی این بیماری بسیار ناهمگون بود و شیوعش از 5 در صدهزار تا 250 درصد هزار متغیر است. ایران، از مناطق با شیوع پایین (5 درصد هزار) محسوب می شود و با توجه به جمعیت ، انتظار می رود حداقل 3000 بیمار مبتلا به M.S در ایران موجود باشد. توزیع سنی، جنسی و دیگر فاکتورهای اپیدمیولوژیک این بیماری در ایران هنوز مورد بررسی قرار نگرفته است و این پژوهش گامی در این راه است. روش بررسی: این مطالعه، روی تمامی بیماران M.S بستری شده در مراکز آموزشی درمانی تهران در سال ،1377 انجام شد. یافته‌ها: یافته های پژوهش نشان داد که اکثر بیماران مورد مطالعه مونث (%63/3 مونث و 36/4% مذکر) بوده و نسبت جنسی زن به مرد حدود 1/74 به یک می باشد. اکثر بیماران متاهل بوده و متوسط سن شروع بیماری 28 سال است. اولین تظاهر بیماری در %49/2 بیماران (بالاترین فراوانی) ضعف اندام و در %33/7 تاری دید و دوبینی و در %32/2 اختلالات حسی بود. نتیجه‌گیری: شایعترین نشانه بالینی، اشکال در راه رفتن بوده و اکثر بیماران با کمک دیگران یا با وسیله کمکی قادر به راه رفتن هستند.

Title: آزمون لب خوانی سارا ساخت، ارزشیابی و اجرا بر روی بزرگسالان دچار ضایعه شنوایی اکتسابی

http://rehabilitationj.uswr.ac.ir/article-1-528-fa.html

هدف: هدف بررسی حاضر ساخت آزمون لبخوانی بزرگسالان فارسی زبان، ارزش یابی آن روی گروه دارای شنوایی هنجار و اجرای آن روی گروهی از بزرگسالان دچار ضایعه شنوایی اکتسابی بوده است. روش بررسی: این بررسی در سه مرحله تهیه مواد، ارزش یابی و اجرای آزمایشی انجام شده است. در مرحله اول با الگو برداری از «آزمون دنور»  آزمونی شامل 20 جمله محاوره ای، روزمره و ساده تهیه شد. در مرحله ارزش یابی آزمون بر روی 105 بزرگسال با شنوایی هنجار اجرا شد. در این مرحله، از دو گزینه (زن ومرد) استفاده شد. در اجرای آزمایشی، 48 فرد دچار ضایعه شنوایی اکتسابی (متوسط/متوسط و شدید/شدید) مورد بررسی قرار گرفتند و آزمون به صورت دیداری صرف و دیداری شنیداری روی آنان اجرا شد. یافته‌ها: بر اساس نتایج به دست آمده، آزمون ساخته شده آزمونی روا و پایا برای ارزیابی توانایی لب خوانی است.

Title: بررسی مقایسه‌ای آزمون تشخیص بین دو نقطه در دست افراد بینا و نابینا در دهه سنی ۱۰ تا ۲۰ سال

http://rehabilitationj.uswr.ac.ir/article-1-529-fa.html

هدف: آزمون تشخیص بین دو نقطه (1) ، یک آزمون حسی عملکردی معتبر و قابل دسترسی است که در ضایعات دست و معاینات نورولوژیک کاربرد دارد و درک آن در ناحیه کورتکس حسی صورت می گیرد. روش بررسی: این بررسی به صورت توصیفی‌تحلیلی و مقطعی انجام گرفت. جامعه مورد مطالعه شامل 240دختر و پسر نابینا و بینا 10-20سال بوده است. آزمون 2PD در ناحیه کف دست و توسط کالیپر استاندارد صورت گرفت. یافته‌ها: دقت 2PD کف دست راست بینایان در مقایسه با نابینایان، بویژه در نیم دهه 10-15 بیشتر بوده است. دقت این تست در پسران بیشتر از دختران است. نتیجه‌گیری: دقت 2PD در نیم دهه 15-20 سال بیشتر از 10-15 سال و همین طور در دست غالب بیش از دست مغلوب به دست آمد و جالب اینکه ، دقت این آزمون در مناطق مختلف یک دست دارای اختلافی به این شرح است: دقت انگشتان شست> اشاره >کوچک> حلقه> وسط.

Title: توانبخشی روانی و جسمی در بیماران مولتیپل اسکلروزیس

http://rehabilitationj.uswr.ac.ir/article-1-530-fa.html

بیماری M.S یک بیماری مزمن دستگاه اعصاب مرکزی است که در آن پوشش میلین به صورت موضعی تخریب می شود. این بیماری یکی از علل مهم ناتوانی در سنین جوانی و میانسال بوده و عمدتا در سنی 20-30 سالگی شایع است. به ندرت در سنین کمتر از 10 سال و بالای 60 سالگی شروع می شود. تخمین زده می شود که حدود 1/1 میلیون نفر در دنیا به این بیماری مبتلا باشند. از آنجا که M.S نواحی مختلف دستگاه عبی را درگیر می کند، می تواند علائم متفاوتی را نیز ایجاد نماید. سیر کلینیکی بیماری از نوع خوش خیم تا فرمهای سریعا پیشرونده متفاوت است و عموما بیماری با حملات رفت و برگشتی مشاهده می شود. حملات اولیه معمولا بهبود می یابند ولی با تکرارحملات، به تدریج عوارض عصبی به صورت دائمی باقی می مانند. علت بیماری M.S ناشناخته است. در مطالعات انجام شده مکانیسمهای اتوایمیون، غفونتهای ویروسی و ژنتیک را دخیل می دانند. عوامل محیطی در شور و تکرار حملات می تواند موثر باشد. شیوع MS در زنان بیشتراز مردان است و از لحاظ جغرافیایی، در نواحی که از خط استوا دورتر هستند بیشتر دیده می شود. از آنجا که بیماران M.S با طیف گسترده ای از علائم گرفتاری دستگاه عصبی مراجعه می کنند، در مقاطعی از دوره درمان دارویی نیاز به اقدامات فیزیوتراپی و بازتوانی دارند. این اختلالات بیشتر به صورت اختلالات شناختی، بینایی، اشکال در صحبت کردن، اشکال در بلع غذا، ضعف، اسپاستیسیتی، اختلالات حسی، مخچه ای، ادراری و مدفوع بروز می کند. این نوشتار، بیشتر روی اقدامات بازتوانی بیمارا M.S تأکید دارد.

Title: موانع روانشناختی در توانبخشی بیماران نورولوژیک

http://rehabilitationj.uswr.ac.ir/article-1-531-fa.html

عوامل روانشناختی از دو طریق بر بیماران نورولوژیک تاثیر می گذارند. یکی مستقیم و از طریق آسیب وارده به نسوج عصبی و دیگر، غیرمستقیم و به  مثابه واکنشی در برابر ناتوانی و معلولیت ایجاد شده. افسردگی: اغلب مانعی در برابر پیشرفت فرایند توانوبخشی است. اصلاح نگرش بیمار به ناتوانی، تقلیل استرس های محیطی و تقویت شبکه حمایتی بیمار همراه با تجویز داروهای ضد افسردگی مانع کناره گیری زودرس او از برنامه های توانبخشی می شود. خستگی مزمن: با کاهش سطح فعالیت بیمار، احتمال دستیابی او را به اهداف تعیین شده توانبخشی کاهش می دهند. حمایت، اطمینان بخشی و گاه داروهای ضدافسردگی مفید واقع می شون. واکنش هیستری: اغلب با ناتوانی ها و بیماری های مزمن همراه شده و مانع ارزیابی و تشخیص صحیح می شود. روش های رفتاری معمولا نتیجه بخش هستند. تغییر خودپنداره: از ویژگی های بارز بیماران نخاعی است. مشاوره و گفتگوی آزاده در مورد تغییر خودپنداره و مشکلات جنسی ضروری است. سایر فرم های بیان جنسی را باید به بیمار آموزش داد. درد مزمن:‌اختلالی ناتوان کننده است. یافته های فیزیکی معمولا بی اهمیت هستند، از این رو گمان می رود عوامل روانشناختی در آن نقش عمده تری داشته باشند. از فیزیوتراپی و کاردرمانی شدید باید اجتناب کرد. داروهای ضدافسردگی در اکثر موارد مفیدند. هیجان پذیری مفرط: معمولا در ضایعات لوب فرونتال  دیده می شود و ناشی از اختلال کارکرد اجرایی است. تمانسوری و عدم بازداری ممکن است به پرخاشگری و رفتارهای نامناسب جنسی منجر شود. تجویز محتاطانه داروهای روانگرا، کاربرد روش های رفتاری، گاه بستری شدن کوتاه مدت در بیمارستان مفید خواهد بود. تغییرات شناختی: در بسیاری از بیماری های مغزی دیده می شود. کمبود توجه، کندی پردازش اطلاعات، اختلال در توانایی های ارتباطی و ضعف حافظه صلاحیت بیمار را برای ورود به برنامه توانبخشی به مخاطره می افکنند. ارزیابی عصب-روانشناختی به تعیین نوع و دامنه آسیب های موجود کمک می کند. از داده های آن می توان برای آموزش اعضای تیم توانبخشی و ارائه رهنمود برای برقراری تعامل موثرتر با بیمار استفاده کرد.
